# Corrective binaural processing for bilateral cochlear implant patients

**DOI:** 10.1371/journal.pone.0187965

**Published:** 2018-01-19

**Authors:** Christopher A. Brown

**Affiliations:** Department of Communication Science and Disorders, University of Pittsburgh, Pittsburgh, PA, 15217, United States of America; Australian Research Council Centre of Excellence in Cognition and its Disorders, AUSTRALIA

## Abstract

Although bilateral cochlear implant users receive input to both ears, they nonetheless have relatively poor localization abilities in the horizontal plane. This is likely because of the two binaural cues, they have good sensitivity to interaural differences of level (inter-aural level differences, or ILDs), but not those of time (inter-aural time differences; ITDs). Here, localization performance is assessed in six bilateral cochlear implant patients when instantaneous ITDs are measured and converted to ILDs, a strategy that results in larger-than-typical ILDs. The added ILDs are corrective, in that they are derived from individual listener performance across both frequency and azimuth, so that they are small where a listener performs well, and increase as performance deviates from ideal. Results show significantly improved localization performance as a result of this strategy, with two of the six listeners achieving levels of performance typically observed in NH listeners.

## Introduction

The criteria for CI candidacy has eased in recent years, and as a result it is becoming more common for individuals with bilateral hearing loss to be fitted with two cochlear implants. The hope is that because these bilateral CI (BCI) users are receiving auditory input in both ears, they may be able to perform binaural tasks such as localization, and improved speech understanding in spatially-separated maskers (ie., solve the well-known cocktail party problem). Results thus far have been disappointing however, as they have shown relatively poor localization abilities, and little benefit in competing-talker scenarios. Of the two binaural cues available to listeners with normal hearing (NH), BCI users generally have better sensitivity to interaural level differences (ILDs), although their ability to localize based on ILDs is still poorer than that for listeners with NH [1–4]. BCI users have shown relatively poor sensitivity to ITDs [5–7], and it is this reduced ITD sensitivity that is likely the limiting factor in their binaural abilities [8].

Although ITDs are present in the acoustic signals captured by cochlear implants (CIs), they are poorly represented in the signals delivered to CI users, because salient ITDs are encoded in the temporal fine-structure (TFS). It is well known that CI signal processing discards most of the acoustic TFS information and instead delivers the more slowly-varying fluctuations, known as the amplitude envelope [9, 10]. Although envelope information alone is often sufficient for good speech intelligibility in quiet [10], CI users perform poorly on speech understanding in competing backgrounds, and other complex perceptual tasks that rely on TFS cues like pitch and ITDs [7, 11, 12], which are poorly represented by CI processing [9, 13].

Improving the binaural representation for CI users is an enormous challenge that is being addressed by a relatively large cross-section of the field on a number of different fronts. For example, there have been efforts to improve ITD sensitivity by better aligning electrodes in frequency using physiological [14] or behavioral [15] procedures. Others have attempted to improve the saliency of ITDs via manipulations of the timing of the electrical pulses [16], or envelope [17]. The possibility of taking advantage of residual acoustic hearing to improve binaural hearing has also been examined [18]. The benefits obtained thus far, however, have been relatively small.

The reliance by BCI users on ILDs has significant implications for localization, speech reception, and other tasks as well. For example, ILDs are generally considered usable above about 2000 Hz or so [19], which means that a significant portion of the frequency spectrum (i.e., most of the energy below 2000 Hz) does not carry a reliable binaural cue for most BCI users [20].

In addition to being inconsistent across frequency, naturally-occurring ILDs are also non-monotonic as a function of azimuth, as illustrated by the well known acoustical “bright spot” [21]. For example, if ILDs are measured at 2 kHz as a function of azimuth at a fixed far-field distance and using a typical human head or acoustic manikin, they will increase as azimuth increases from zero until about 55°, at which point the ILDs will begin to decrease with a local minimum at about 90°, where the ILD is about equal to that at 25°. There is evidence from both animal [22] and modeling [4] studies that source location estimation based on ILDs can be disambiguated by across-frequency pattern analysis, although it is unclear whether BCI users possess this ability. However, given the limited number of available CI channels [23], and the relatively poor localization abilities exhibited by BCI users [24], this possibility seems unlikely. Given that BCI users rely on ILDs, the poor localization performance shown by BCI users thus far [25] is not surprising.

**Fig 1:**
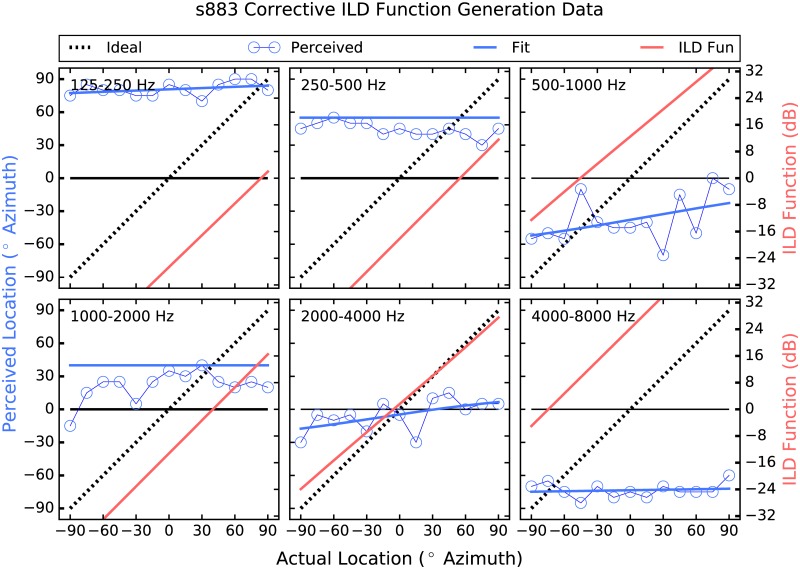
Corrective ILD generation data for participant 883. The six inset panels each represent a different frequency band, with cutoff frequencies indicated in the upper left corner of each inset panel. In each inset panel, the dashed black line represents ideal performance, the open blue circle markers depict perceived location plotted as a function of source location, and the heavy blue plot is a sigmoid fit to the localization data. The red functions are the corrective ILD functions for each band, which are the respective differences between ideal and the fit sigmoids, are expressed in dB, and use the y-axes to the right.

**Fig 2:**
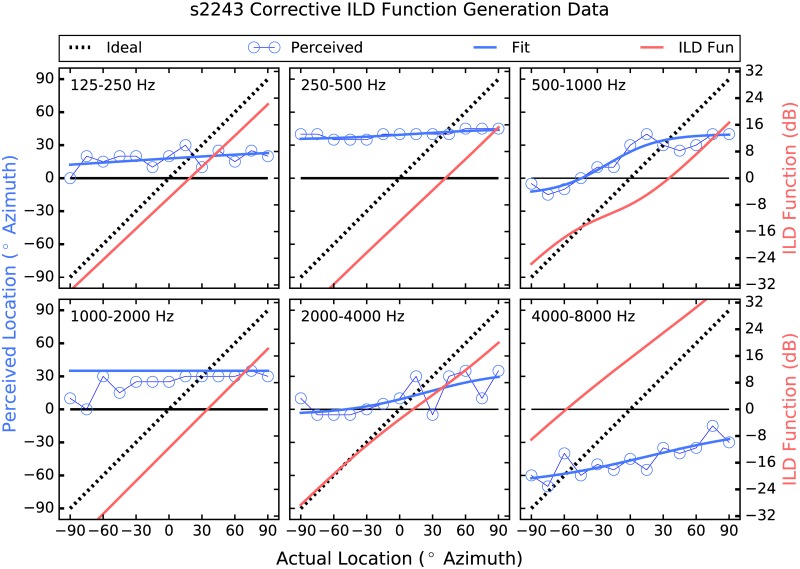
Corrective ILD generation data for participant 2243. The layout is identical to that of Fig 1.

**Fig 3:**
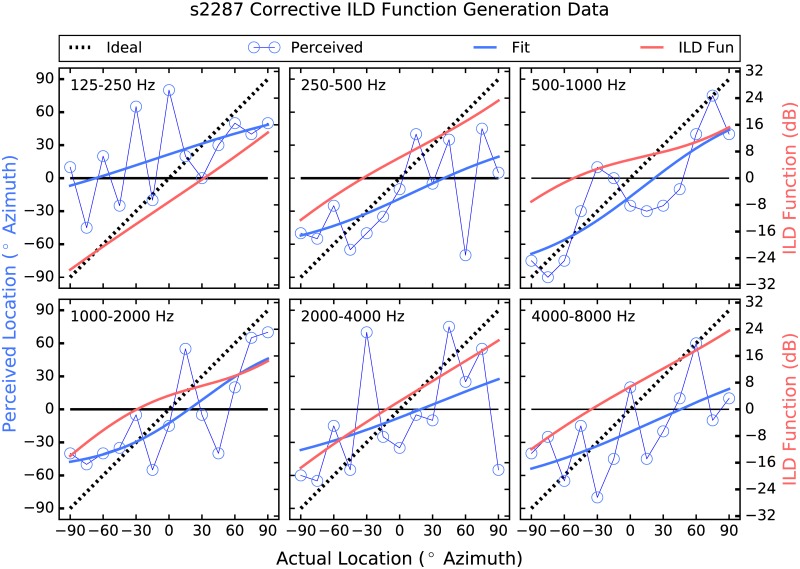
Corrective ILD generation data for participant 2287. The layout is identical to that of Fig 1.

**Fig 4:**
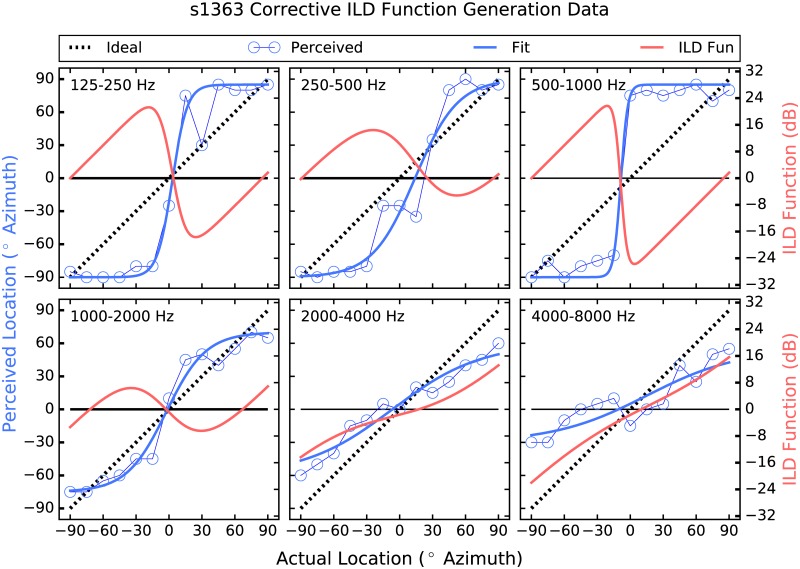
Corrective ILD generation data for participant 1363. The layout is identical to that of Fig 1.

**Fig 5:**
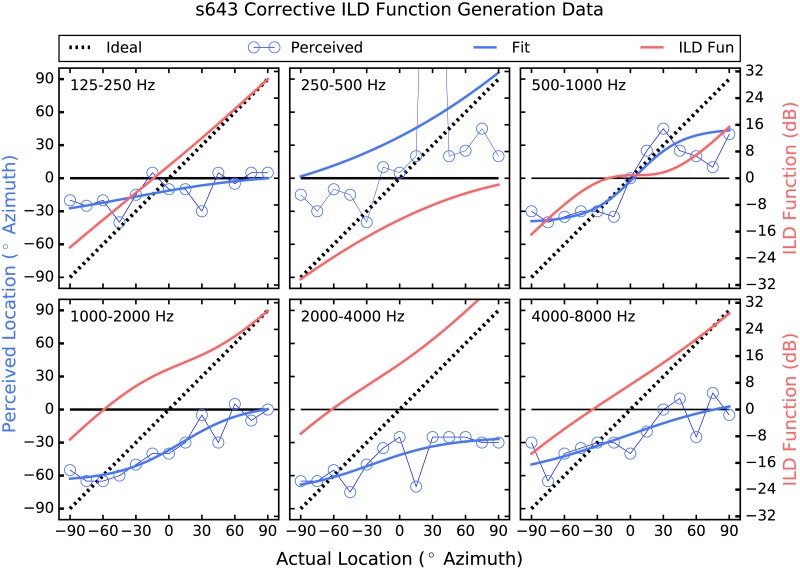
Corrective ILD generation data for participant 643. The layout is identical to that of Fig 1.

**Fig 6:**
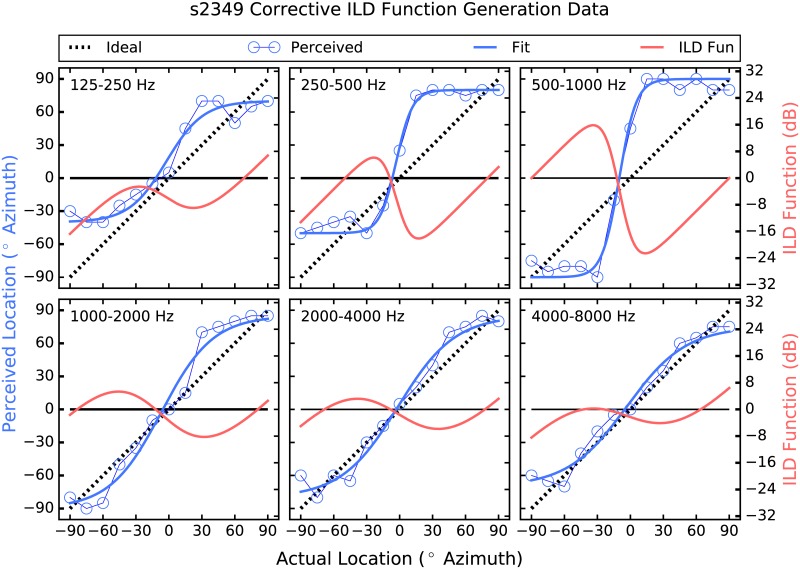
Corrective ILD generation data for participant 2349. The layout is identical to that of Fig 1.

The strategy employed here attempts to address these shortcomings by providing larger-than-normal ILD cues [26]. Instantaneous ITDs are estimated, which are present acoustically but not commonly deliverable to the user, and are converted to ILDs, which are not robust enough in all frequency regions to be useful but have been shown to be perceivable irrespective of frequency when made large enough [27]. Further, a corrective approach is taken in which the size of the ILD applied in each frequency band and at each location is determined by each listener’s performance there. Thus, if a listener shows good localization performance in a particular frequency band and at a given location, then the strategy does little, and the size of the ILDs applied increases as performance deviates from ideal (see Figs 1–6). The goal is to provide an ILD that delivers a more consistent percept across frequency [28] by increasing the magnitude of the cue where and by as much as is needed.

## Method

### Participants

Six adult BCI listeners participated, all of whom had at least 24 months of experience with two CIs. Please see Table 1 for participant demographics and Table 2 for device information. This research involved human subjects, and was approved by the University of Pittsburgh Institutional Review Board (IRB# REN16080059). All participants provided written informed consent prior to participating in the study.

**Table 1:** There is one listener per row. Data include Gender, Age, the Etiology of the hearing loss, and the number of years of experience with the Left and Right CI.

Subject	Gender	Age	Etiology	Years CI; Left	Years CI; Right
883	Female	70.9	Unknown	8.8	16.0
2243	Male	65.6	Noise Exposure	2.0	2.7
2287	Male	55	Unknown	8.6	14.7
1363	Male	62.1	Unknown	4.6	4.1
643	Male	63	Noise Exposure	9.8	8.8
2349	Female	54.7	Unknown	2.5	2.0

**Table 2:** There is one listener per row. Data include the manufacturer of participant’s CIs, the CI models the participant uses day-to-day, and the models used in the study. For the MedEl patient, the cutoff frequencies of the four lowest processing channels, in which FSP takes place, are listed beside the left and right Strategies.

Subject	CI Make	CI Model; Daily	CI Model; Testing	Strategy; Left	Strategy; Right
883	AB	Harmony	Harmony	HiRes-P w/ Fidelity 120	CIS
2243	AB	Naida	Harmony	HiRes-P w/ Fidelity 120	HiRes-P w/ Fidelity 120
2287	AB	Naida	Harmony	HiRes-S w/ Fidelity 120	HiRes-S w/ Fidelity 120
1363	AB	Neptune	Harmony	HiRes-S w/ Fidelity 120	HiRes-S w/ Fidelity 120
643	AB	Naida	Harmony	HiRes Optima-S	HiRes Optima-S
2349	MED-EL	Sonnet	Sonnet	FSP (100, 208, 352, 545, 806 Hz)	FSP (100, 208, 352, 545, 806 Hz)

### Procedure and stimuli

Each sat in an echo-reduced listening room (RT60 = 97 ms) that contained a 13-loudspeaker array arranged in an arc in the front hemifield 1.67 m away from the listening position, and at the height of the seated listeners’ pinnae. The loudspeakers were positioned from −90° to +90° with 15° of separation between each. Thus, all possible loudspeakers produced sound. The average presentation level was 65 dB SPL.

The stimuli consisted of 500-ms broadband Gaussian noise bursts that were digitally-generated at a 44.1 kHz sampling rate (22 kHz bandwidth), with 20-ms raised-cosine onset and offset ramps applied. Noise was chosen over speech stimuli because the flat frequency spectrum afforded audible sound on each trial, even after bandpass filtering in the corrective ILD function generation phase of the study. A random level rove of ±3 dB was applied from trial to trial to minimize the effects of level cues that might arise as a result of differences across loudspeakers. Each run consisted of 13 trials, with each loudspeaker location randomly selected without replacement. Each condition was run 3 times, and performance was averaged across the three runs. The subjects’ task was to identify the loudspeaker that presented the noise, and responses were made via computer keypress. Subjects were told that they were free to rotate their head while choosing a loudspeaker, but that they should face the loudspeaker directly in front of them when a trial began, although this was not enforced.

### Conditions

In one condition (“Unprocessed”), listeners used their own devices including the device microphones, and their everyday program, and no research hardware or signal processing was utilized. In another condition (“Processed”), the research platform was used, with the experimental signal-processing strategy described below.

## Signal processing

The research platform consisted of two high-fidelity $\frac{1}{4}$” omni-directional electret condenser microphones to capture sound, mounted on custom earhooks so that they faced forward and were positioned just above and in front of the pinnae. Real-time signal processing took place on an Android phone, using custom-written code. 20-ms processing windows were used, with no overlap, at a sampling rate of 48 kHz. Sound was delivered to each CI using the devices’ accessory cables. In one case (S2349), it was not technically possible to disable the processor microphones, so device microphone sensitivity was decreased as much as possible, which achieved a 90/10 aux-in/microphone mix ratio. The other five patients were all Advanced Bionics users; one patient used Harmony processors, one Neptune, and three used Naida processors. For these participants, their clinical maps were placed onto Harmony Research processors for testing, which allowed the use of clinical programming software to disable the processor microphones without the need to make modifications to their devices. No other changes to aspects of CI processing, such as automatic gain control, were made.

To generate the corrective ILD functions, localization was measured using Gaussian noise that was filtered into one of six contiguous one-octave-wide bands between 125 Hz and 8 kHz prior to presentation from a loudspeaker. During this phase of the study, users’ device microphones were disabled, and the research platform described above was used to deliver signals to the CI devices otherwise unprocessed. A corrective ILD function was generated for each frequency band, by fitting a sigmoid function to the psychometric data generated, which was then subtracted from ideal performance. See Fig 1, in which the corrective ILD functions use the Y axis to the right. It can be seen that when performance is near ideal (when the sigmoid is near the dashed line), the corrective ILD function is near zero, and as performance deviates from ideal, the prescribed ILD becomes larger. The corrective ILD functions were 1501 in length, to cover the range of possible ITDs (integer values, in samples, between ±750 *μ*sec) used in the study. 750 *μ*sec was chosen as the maximum ITD to use because it was slightly larger than the maximum ITD measured when using an acoustic manikin. A maximum ILD of 32 dB was chosen for similar reasons.

In the “Processed” condition, the research platform filtered the incoming audio data from the left and right channels into 6 contiguous 1-octave-wide bands between 125 Hz and 8 kHz. In each band, a sliding lag cross-correlation was used to estimate the ITD, which was the delay that produced the largest correlation. A maximum possible ITD of ±750 *μ*sec was used. An ILD was then generated using the estimated ITD as an index into the corrective ILD function. ILDs of up to 32 dB were applied via attenuation at the far ear, and any amount beyond 32 dB was applied via gain at the near ear. For example, if an ILD of 38 dB was called for, the signal at the far ear was attenuated by 32 dB, and the near-ear signal was increased in level by 6 dB. ILDs larger than 32 dB occurred only occasionally, when the difference between the actual and perceived source locations was greater than 90°. This occurred in three frequency bands for S883, as can be seen in panels 125–250, 500–1000, and 4000–8000 Hz of Fig 1a, in which the Corrective ILD functions extend beyond the bounds of the panels. This also occurred for S2243 in bands 250–500, 1000–2000, and 4000–8000 Hz, and for S643 in band 2000–4000 Hz. No other participant perceived a location that was, on average, further than 90° from the actual location.

## Results and discussion

**Fig 7:**
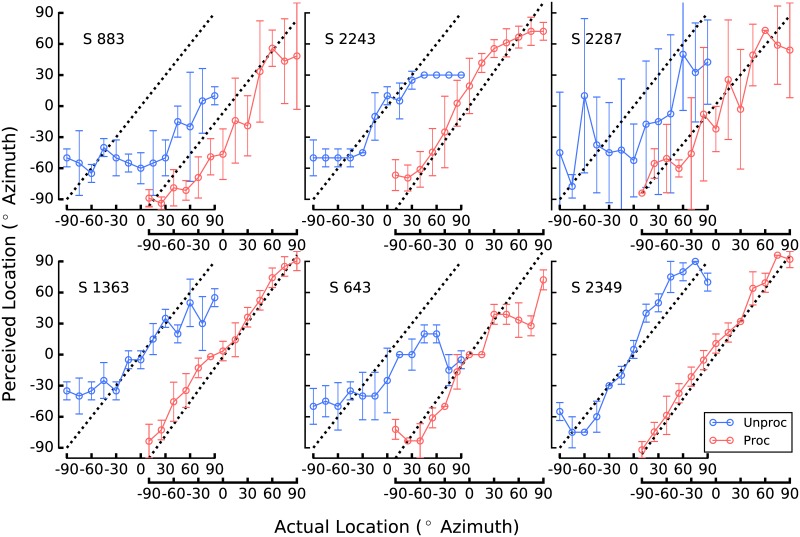
Localization performance for each participant. Each of the six participants are each represented in a separate panel. The blue open circles represent performance in the Unprocessed condition, the red open circles represent performance in the Processed condition, and error bars represent standard deviations. The X axes are staggered within each panel to facilitate comparison and to conserve space.

Fig 7 shows localization performance by each subject in both conditions. The abscissae are staggered for each subject, to facilitate comparison and to conserve space. Root-mean-square (RMS) error was computed for each listener in each condition. This measure was used as the dependent variable in a related-measures t-test, which revealed a significant difference in performance between the two conditions, t = 4.8, p = .0003. This result indicates the corrective processing provided a statistically significant improvement to localization accuracy over the listeners’ typical configurations. Across listeners, average RMS error was 31.0° in the Unprocessed condition, and 12.8° in the Processed condition.

Mean RMS error in the Unprocessed condition of the current study (31°) is on par with that reported in the literature, which has varied somewhat across studies between 20 and about 30° [1, 2, 25]. Recent studies have reported mean RMS error for listeners with NH localizing broadband noise to be about 6°, with the poorest performers exhibiting about 12° of error [1, 19]. Average performance in the Processed condition (12.8° of error) is approaching this range, with two listeners performing similar to the reported NH-listener average (S 1363 at 7.3° and S 2349 at 5.2° of error). Table 3 depicts RMS error, in degrees azimuth, for the six participants in the Unprocessed and Processed conditions.

**Table 3:** There is one listener per row. Data show RMS error in degrees azimuth for the Unprocessed and Processed conditions.

Subject	Unproc Error	Proc Error
883	40.2°	15.3
2243	26.1	14.0
2287	36.7	17.6
1363	26.9	7.3
643	38.4	17.6
2349	17.5	5.2

### High-frequency processing

The processing used in the current study was applied to octave-wide frequency bands that extended up to 8 kHz. This choice may benefit from some discussion since it is generally acknowledged that ITDs are ‘usable’ only below 1.4 kHz [29]. This is true for pure tones, for which the short wavelengths associated with higher frequencies limit the size of the delay that can be perceived in ongoing signals (longer delays can be perceived at signal onsets). Transients and other aperiodic signals like noise, however, do not necessarily have the same limitations. When it is considered that much of the high-frequency energy in speech is derived from sibilants and plosives, both of which are aperiodic, it is reasonable to think that successful high-frequency ITD encoding is possible.

**Fig 8:**
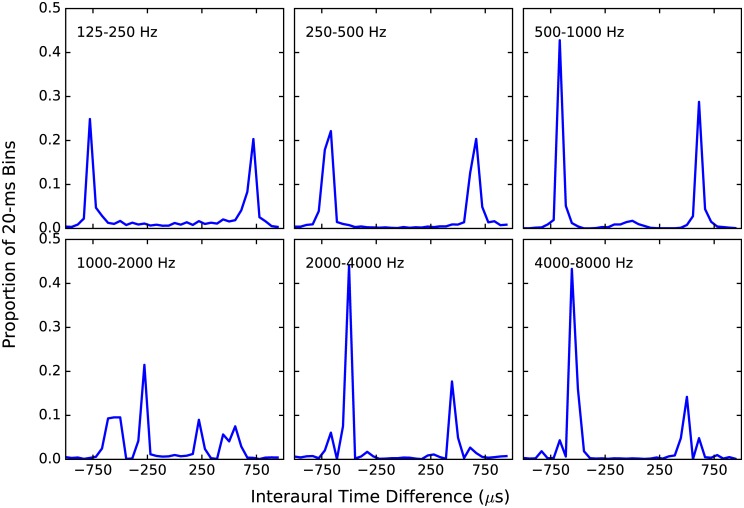
ITD frequency histograms for various frequencies. Acoustic analysis was conducted on 50 pairs of sentences (about 2.5 minutes of data) produced by different female talkers, in which head-related impulse responses were applied to each such that one was 60 degrees to the left, and the other 60 degrees to the right. The sentences were then combined, filtered into six one-octave wide frequency bands, and an ITD was then estimated every 20 ms in each band. Each plot represents the proportion of occurrence of a given ITD among the time bins. Each panel shows acoustic analysis data for a particular frequency band.

Fig 8 confirms this assumption, and depicts frequency histograms of the estimated ITDs in 20-ms time bins for two concurrent talkers when non-individualized head-related impulse responses (HRIRs) were applied to each such that one was 60 degrees to the left of midline, and the other was 60 degrees to the right. Fifty sentence tokens of each female talker were used, or about 3 minutes of audio data. Each panel of Fig 8 shows data for a different frequency band. It can be seen that in most frequency bands, ITDs tend to cluster around one of two modes even in the 4–8 kHz band. The poorest spatial representation occurs in the 1–2 kHz band, which appears to have four distinct modes. It is unclear why this pattern is observed, although it was replicated using both 50 different tokens from the same two talkers, and 50 tokens each from 2 male talkers. Despite this unexplained result, this analysis indicates that reliable ITD estimation is possible even in this relatively high frequency region, at least for the talkers used in this analysis.

Another unexpected outcome from this acoustic analysis was the magnitude of the ITDs measured. For example, it can be seen that in the 125–250 Hz band, the modal ITD was around 750 us. Given that 750 *μ*s was the largest ITD estimated in the current study, and that the analysis presented in Fig 8 was generated with non-individualized HRIRs from sources locations of ±60°, it seems clear that there is a distinct possibility that some ITD estimates in the current study were not as veridical as they may otherwise have been, particularly at more lateral source positions, and in the lower frequency bands. When ±90° source locations were used and the analysis re-run, modal ITDs in the lowest band were around 1000 *μs*. However, because each listener has a potentially different head size, quantifying this source of error would require making HRTF measurements of each source location for each listener, which is not feasible after the fact. In any event, the most likely result is that in the moments of time when the actual ITD exceeded the maximum measured ITD, the applied ILD would be smaller, and probably more variable than it otherwise would have been.

In retrospect, microphone placement may have also exacerbated this problem. In the Processed conditions, subjects wore the custom ear-hook microphones in addition to a behind-the-ear (BTE) speech processor, and in a case or two the temples of eyeglasses. It was thus sometimes cumbersome to securely and comfortably mount the custom earhook microphones onto the pinnae of the listeners. In some cases, the most comfortable and secure approach was to mount the ear-hook mics to the outside of the BTE processor, and depending on placement, the gap between the custom mics and the side of the head is estimated to have been as much as 3/8” on either side. This may have made the measured ITDs in the study larger still, although custom microphone earhook placement was not documented during testing.

A related issue is the pre-emphasis filtering that is applied by CI devices at the front end processing stage, which have a high-pass characteristic. For example, Advanced Bionics devices use a pre-emphasis filter in which the energy at 100 Hz is attenuated by about 30 dB relative to the energy at around 3 kHz (the half-power point is around 1400 Hz). This has the effect of enhancing energy in the high-frequency region, and may lead users to weight cues in that frequency region more heavily. This effect was further emphasized by the use of Gaussian white noise in the current experiment, as opposed to pink or speech shaped noise, which have low-pass characteristics.

### Individual differences

One of the more unexpected results of the current experiment is the seemingly disparate psychometric functions generated in the band-pass conditions. For example, patient 883 (Fig 1a) shows very little ability to discriminate source locations in any of the band-pass conditions. In fact, the most striking aspect of this patient’s data is its frequency-specific nature, observable in Fig 1. That is, when localizing noise filtered between 125 and 250 Hz, patient 883 hears all source locations almost all the way to the right. Conversely, noise filtered between 4 and 8 kHz is heard almost all the way to the left, regardless of source location. Frequency bands between those two are heard nearer midline. The perceptual stability of this pattern of results was confirmed a week later when Patient 883 was brought back in to the lab and tested again in the band-pass conditions. This pattern of performance could be explained by asymmetries in neural survival or depth of insertion of the electrode arrays, poor clinical fitting, or some combination. Even when loudness is interaurally balanced (as from a good clinical fitting), an ILD of zero dB does not always produce a centered image [30, 31].

Patient 1363 (Fig 1d) shows a different pattern which is non-the-less relatively frequency specific. For the two highest frequency bands, the psychometric functions are more or less linear, if somewhat compressed. Specifically, in these frequency regions this participant does not perceive any locations beyond about ±60° or so. This may be due to the amount of compression required by CI devices, as well as the non-monotonic nature of the ILD cue. In the four lower frequency bands, on the other hand, patient 1363 tends to hyper-lateralize. For example, in the 500–1000 Hz band, sound sources appear to this listener as either near +90 or −90°, with no intermediate perceived locations. In the lowest frequency bands, the strategy delivered contra-ILDs in which the applied ILDs would, on their own, have indicated a source location to the contralateral side of the actual source. This is an intriguing result, and warrants further study. It has been suggested that across-frequency pattern analysis can be used to derive source location from the ILD cue [4, 22]. On the other hand, the pattern of results from patients 1363 and 2349 suggest that broadband localization may be the product of relatively simple across-frequency integration of the ILD cue, at least for these two patients. The relatively crude spectral representation that BCI listeners receive makes this a reasonable possibility. It could also be that the low-frequency bands are not perceptually weighted as heavily as the higher-frequency bands by these participants, possibly due to pre-emphasis filtering etc. as described above. In any event, the low-frequency hyper-lateralization by these two participants may be related to loudness recruitment and the omni-directional microphones used.

The interaction between the currently proposed strategy with particular CI coding strategies warrants further study. For continuous-interleaved strategies (CIS) such as with Advanced Bionics devices and in the higher-frequency channels of the FSP family of MedEl strategies, each channel is enabled on each cycle, which may make their effects easier to anticipate. The effects of peak-picking strategies, on the other hand, are harder to predict. MedEl’s FSP strategies use peak-picking in the lower 4 channels, while devices from Cochlear Corp use peak picking across the entire range of channels. Peak picking strategies are also known as n-of-m strategies, in that they analyze the levels of m channels on each cycle, and choose the n channels with the highest amplitudes, which are the only channels enabled on that cycle. Thus, a number of channels are off on any given cycle. This has potentially significant implications for ILD perception by BCI users who use peak-picking strategies. For example, infinite ILDs are frequently delivered when the ipsilateral level at a given frequency region is high enough to be selected while the corresponding contralateral level is too low and that channel is not selected. In these cases, the frequency-specific ILDs may be made larger than what occurs naturally, but there may be more variability across frequency in the ILD cue. Making the ILD larger than natural, as the currently proposed strategy does, may contribute to this problem. If high-frequency ILDs are, in fact, weighted more heavily than those in the low-frequencies, however, this problem may not be as severe with the FSP strategies of MedEl devices, where peak-picking is used only in the lower four frequency bands. The cutoff frequencies of the 4 low-frequency (FSP) channels of the MedEl patient (Subject 2349) are listed in the “Strategy” columns of Table 2. It will be interesting to see the benefits of the proposed strategy with users of devices from Cochlear Corp., which use peak-picking across the entire frequency range. No users of Cochlear devices participated in the current study because difficulties in disabling the device microphones (a safety feature) made it difficult to ensure that listeners heard only the processed sound. Experiments examining the interaction between the proposed strategy and those from the different manufacturers are planned.

### ILD magnitude before and after processing

**Fig 9:**
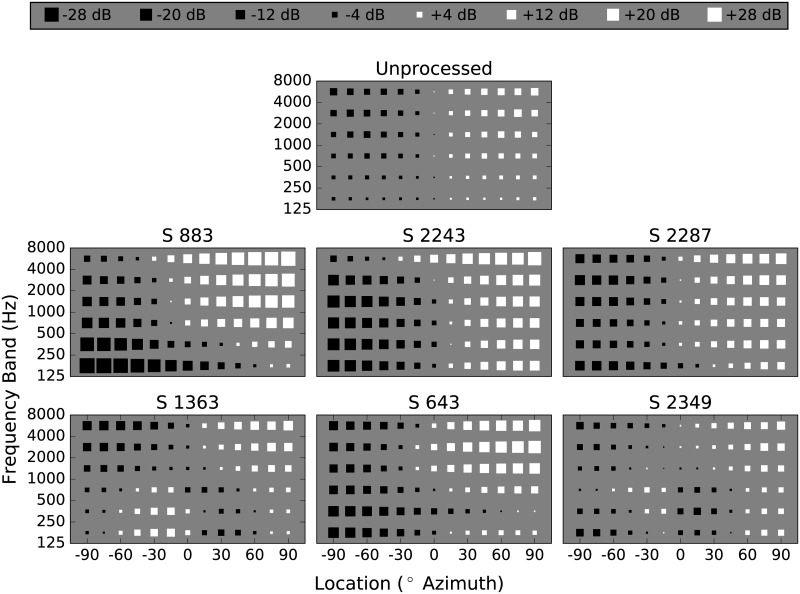
Pre- and post-processed ILD estimates as a function of azimuth for each participant. Acoustic analysis is of 500-ms broadband Gaussian noise, recorded from source locations at +/-90 degrees in 15-degree steps, using omnidirectional 1/4” microphones mounted on an acoustic manikin facing forward just above the auricles, as was done during testing. The top panel depicts ILDs in the unprocessed condition, and the the lower six panels each represent ILDs after processing using the six listeners corrective ILD functions, one panel per listener. In each panel, maker size represents ILD magnitude in each of the six frequency bands used in the study as a function of source location. Black markers represent apparent source locations to the left, and white markers to the right.

To gain some insight into the actual ILDs that were present after processing, acoustic analysis was performed as follows. First, 500-ms broadband Gaussian noise was recorded from the 13 locations used in the current study, using the earhook microphones described in the Methods section, mounted on an acoustic manikin at the listening position. These recordings were either analyzed as is (Unprocessed), or after processing with the algorithm described, and using the corrective ILD functions generated by each of the participants. The analysis consisted of filtering the noise into the six 1-octave wide frequency bands used in the current study, and computing an ILD in each band. Fig 9 shows the results of this analysis, with the top panel depicting the Unprocessed stimuli, and the lower six panels the stimuli processed with the algorithm, one for each participant. In each panel, frequency band is presented on the Y axis, and source location is on the abscissa. The size of the marker represents the magnitude of the ILD in that frequency band and at that location, and black markers indicate an apparent source location to the left of midline, while white markers indicate apparent sources to the right.

The largest ILD measured in the Unprocessed conditions was 7.5 dB. Although this is somewhat smaller than the ILDs that typically occur in the ear for listeners with NH at these frequencies, it is on par with the ILDs reported in the literature for a behind-the-ear microphone placement [32]. The largest ILD measured in any of the processed conditions is 28.5 dB, for subject 883. This value appears lower than expected, given the ILD functions for this listener. One potential cause of this is the possible smaller-than-expected applied ILDs that might occur when the measured ITDs are greater than anticipated, and particularly if they are greater than the maximum ITD chosen for this study.

Regardless of the cause, if the applied ILDs would have been too small, this would have been observable in the localization data in the form of compressive, sigmoidally-shaped localization functions. This might have been addressed by increasing the maximum ILD parameter of the algorithm. Nevertheless, more work will be needed to address how larger-than-typical ILDs drive perceived location.

All participants showed reduced RMS error when provided with the corrective ILD processing strategy. But four of the six subjects still showed performance that does not appear to be in the range exhibited by typical NH listeners. One interesting possibility for BCI users like these is that the applied ILDs be made larger still, and users be provided exposure over time to the very large ILDs. The acute outcome would likely be that these listeners would hyper-lateralize, given the very large ILDs. But it is possible that if they are exposed over time to the large ILDs, they may be able to adapt to them and re-map their perceptual space to account for them. In such a scenario, it may be that localization performance eventually becomes even better with very large ILDs. That is, using very large ILDs might increase the available dynamic range of ILDs across the horizontal plane, possibly improving acuity when expressed in degrees azimuth. On the other hand, the maximum realizable ILD will be constrained by the input dynamic range of the devices. Thus, there are technical limitations to what is possible.

Nevertheless, the approach outlined here, combined with the portable real-time platform offers an intriguing opportunity to explore binaural plasticity, and the possibility of adaptation to altered ILD cues particularly. There are a few such experiments in the literature, but none have directly examined ILD adaptation, in part because of the technical challenges associated with implementation. For example, Young constructed a ‘pseudophone’ which positioned acoustic collectors on either side of the head, and used tubes to deliver the sound collected from each to the respective contralateral ears of the listener [33]. The author wore this passive instrument continuously for 85 hours, with the goal of observing if there was any adaptation to the reversed binaural cues, but found none. However, given that modern ear plugs typically provide 20–30 dB of attenuation [34], an amount that leaves signals audible when produced at typical conversational levels, it is unlikely that the apparatus used by Young effectively isolated him from the original, veridical sound. This would mean that he likely received a mixture of the two sounds, which makes interpretation of the results of the study difficult.

Other investigators have looked at aspects of adaptation to spatial cues, with most focusing on spectral cues. For example, adaptation has been shown for vertical localization when spectral cues were altered by applying molds to the pinnae of participants [35], and for reducing front-back confusions in the horizontal plane when HRTFs were non-individualized [36]. Several studies have demonstrated adaptation to changes in the ITD cue in a horizontal-plane localization task by implementing a constant interaural delay using bilateral hearing aids programmed with asymmetrical digital delays [37], or by placing participants under water, where the speed of sound is greater than in air, and thus the ITD cue is reduced for given source angles [38]. No studies were found that directly examined adaptation to altered ILD cues.

It appears from these results that the binaural representation of at least some individuals in this population can be quite complex, idiosyncratic, and non-monotonic. It is unknown at this time what the source or sources of this variability are, although there are a number of likely candidates, including their reliance on ILDs [1], and the speech coding strategies of the devices [3, 39]. Another likely factor involves the interface between individual electrodes of each device and the regions of auditory nerve they each stimulate. In a normal healthy auditory system there is precise interaural frequency alignment, which is required for good binaural fusion. That is, if components to each ear stimulate neurons in different tonotopic regions, then they will likely not be perceived as a single auditory object, and no binaural fusion will occur. For example, if there is an ILD present between two such components, it may not contribute to a spatial percept, but rather one component may be perceived as being lower in level than the other, in addition to being different in frequency. However, this appears to be less of a problem for BCI users in particular [15], perhaps because the relatively course frequency resolution and broad spread of current [40] may make electrode alignment somewhat less critical for ILD processing, although this possibility would need to be established empirically.

## Conclusion

The proposed corrective processing appears able to provide benefit for both above-average (S1363, S2349) and below-average (S883, S643) performers, with the performance of some listeners approaching that of NH. The results of the current study demonstrate that although naturally-occurring ILDs alone are often not sufficient for good localization by BCI users, applying them in a corrective way as was done in the current study can lead to significant improvements in localization for this population.

## Supporting information

S1 File Localization data. The data file is in comma-separated-value text format. Columns are participant number, condition, and actual and mean perceived location, in degrees azimuth. Data for the Unprocessed and Processed conditions are the data for the main experiment, and the other conditions are the band-specific conditions used for generating the corrective functions, where eg., band_250_500 refers to the condition in which the high-pass and low-pass cutoff frequencies were 250 and 500 Hz.
(CSV)
